# Innovations in Camera Trapping Technology and Approaches: The Integration of Citizen Science and Artificial Intelligence

**DOI:** 10.3390/ani10010132

**Published:** 2020-01-14

**Authors:** Siân E. Green, Jonathan P. Rees, Philip A. Stephens, Russell A. Hill, Anthony J. Giordano

**Affiliations:** 1 Department of Anthropology, Durham University, Durham DH1 3LE, UK; r.a.hill@durham.ac.uk; 2 Conservation Ecology Group, Department of Biosciences, Durham University, Durham DH1 3LE, UK; jonathan.p.rees@durham.ac.uk (J.P.R.); philip.stephens@durham.ac.uk (P.A.S.); 3 The Society for Preservation of Endangered Carnivores and Their International Ecological Study (SPECIES), Ventura, CA 93006, USA; species1@hotmail.com

**Keywords:** camera trapping, citizen science, artificial intelligence, engagement, camera traps, public awareness, data processing, conservation technology

## Abstract

**Simple Summary:**

Camera traps, also known as “game cameras” or “trail cameras”, have increasingly been used in wildlife research over the last 20 years. Although early units were bulky and the set-up was complicated, modern camera traps are compact, integrated units able to collect vast digital datasets. Some of the challenges now facing researchers include the time required to view, classify, and sort all of the footage collected, as well as the logistics of establishing and maintaining camera trap sampling arrays across wide geographic areas. One solution to this problem is to enlist or recruit the public for help as ‘citizen scientists’ collecting and processing data. Artificial Intelligence (AI) is also being used to identify animals in digital photos and video; however, this process is relatively new, and machine-based classifications are not yet fully reliable. By combining citizen science with AI, it should be possible to improve efficiency and increase classification accuracy, while simultaneously maintaining and promoting the benefits associated with public engagement with, and awareness of, wildlife.

**Abstract:**

Camera trapping has become an increasingly reliable and mainstream tool for surveying a diversity of wildlife species. Concurrent with this has been an increasing effort to involve the wider public in the research process, in an approach known as ‘citizen science’. To date, millions of people have contributed to research across a wide variety of disciplines as a result. Although their value for public engagement was recognised early on, camera traps were initially ill-suited for citizen science. As camera trap technology has evolved, cameras have become more user-friendly and the enormous quantities of data they now collect has led researchers to seek assistance in classifying footage. This has now made camera trap research a prime candidate for citizen science, as reflected by the large number of camera trap projects now integrating public participation. Researchers are also turning to Artificial Intelligence (AI) to assist with classification of footage. Although this rapidly-advancing field is already proving a useful tool, accuracy is variable and AI does not provide the social and engagement benefits associated with citizen science approaches. We propose, as a solution, more efforts to combine citizen science with AI to improve classification accuracy and efficiency while maintaining public involvement.

## 1. Introduction

Camera traps, also known as trail cameras or game cameras, are devices used remotely to record wildlife activity. They can be left in the field for long periods of time and can be activated to take photos or videos by a variety of mechanisms including weight plates and both active and passive infra-red sensors. The past 20 years have seen a rapid expansion of camera trap use [1,2]. So much camera trap footage is now collected that analysing it can become a major challenge for researchers [3]. Both citizen science and use of Artificial Intelligence (AI) are also rapidly growing fields and have been put forward as potential solutions to this problem [3,4,5,6,7]. Citizen science and AI approaches have their own advantages and disadvantages, but when combined have great potential to play a pivotal role in the future of camera trap research. However, in order to successfully incorporate citizen science and use of AI into camera trap research, an understanding of all three approaches is needed. There are large bodies of literature focussing on these individual elements separately but little that brings all three together. Here we provide an introduction to these approaches and describe current methods being used to integrate camera trapping with citizen science and AI. This review uniquely highlights the ways in which these methods could be combined and provides a background to these topics, with the aim of encouraging researchers to consider if and how their work could benefit from integrating camera trapping, citizen science and AI.

The origins of the camera trapping technique extend as far back as the 1890s [8]. George Shiras developed a technique for photographing wildlife by incorporating a tripwire that an animal triggered; this gained him recognition for introducing a novel approach to wildlife photography [9]. As Shiras was not a professional scientist, the origin of camera trapping to record wildlife began in the public domain. Even during early camera trap research, it was obvious that the images generated had the potential to serve as a powerful public engagement tool with Griffiths, M.; van Schaik, C.P stating in 1993 that: “People’s minds and hearts are reached best through visual means, and an important spin-off of the photo census work is that it boosts conservation through publicizing the richness of a reserve” [10] (p. 134). It was also apparent early on that not only scientists were interested in camera-trapping, with hunters wishing to record game too. This attention was significant for the development of camera trap technology as the game hunting community created a market demand for the product much greater than that from scientists alone [11,12,13]. The consequence of this was that brands focussed on retailing hunting related products and accessories invested in developing camera traps. Many manufacturers now produce cameras popular with both game hunters and wildlife researchers, and a wide range of brands and models are available [2]. There is also an increasing market from wildlife enthusiasts wishing to record local wildlife for interest and enjoyment, and camera traps are now readily available to purchase in non-specialist stores such as mainstream supermarkets.

From the very start, the strong connection between camera trapping and the public highlighted the potential of camera trapping as an engagement tool. Yet, public engagement has not always been evident in camera trapping. More recently, the value of public engagement is being increasingly recognised, as indicated by the increasing amount of camera trap research involving citizen science.

## 2. Citizen Science

Citizen science is a general term normally used to describe scientific work that has been undertaken by members of the general public, often, though not always, in collaboration with professional scientists or institutions [14,15,16,17]. Ongoing debate centres on the most appropriate terminology for this approach, with concerns around inclusivity and cultural differences leading to different interpretations and preferences [18,19]. Some may find the term ‘citizen’ exclusionary, because the apparent link to legal citizenship of a particular state or nation can be a sensitive issue [18]. Others object to the implied distinction between ‘professional’ and ‘other’ scientists. Alternative terms for public involvement in research include public participation in scientific research, participatory action research, crowdsourcing and community based research, with subtle differences in how these terms are applied [18,19,20]. An awareness of such issues is important for those wishing to engage the public in their research, to avoid offending or disenfranchising those they are aiming to engage. We use the term ‘citizen science’ as it is a widely recognised term but wish for it to be interpreted in a broad and inclusive way.

Various broad categories for types of citizen science projects have been described, although not all projects will fit neatly into one of them. Examples of these categories are: (1) Contributory, where projects are “generally designed by scientists and for which members of the public primarily contribute data”; (2) Collaborative, where projects are “generally designed by scientists, and for which members of the public contribute data but may also may help to refine project design, analyse data, or disseminate findings”; and (3) Co-created, where projects “are designed by scientists and members of the public working together and for which at least some of the public participants are actively involved in most or all steps of the scientific process” [20] (p. 11). (4) Contractual projects, where a community seeks out professional researchers to conduct a specific investigation; (5) Collegial contributions, where non-credentialed individuals have conducted independent research recognized to some extent by institutionalised science [19] and; (6) a category which does not involve professional scientists at any stage and all stages of work are carried out by the general public [21].

Involvement in citizen science projects has led to increased knowledge among volunteers of the topics being researched [15,22], as well as changes in behaviour [23,24]. Changes seen in nature-based citizen science projects include increased advocacy for species, creating wildlife-friendly habitats [25,26] and increased likelihood of discussing conservation-related issues [22]. Citizen science can also provide a sense of purpose and community, and allow people to develop new skills and knowledge [27,28]. Participating in conservation-based citizen science can help establish a link to nature for people of all ages. Taking too much leisure time can make people feel guilty or lazy and, in some cases, people have described participating in citizen science as a good excuse to slow down and enjoy nature, in a way that would otherwise have made them feel guilty [28,29]. This increased engagement with the natural world can have a variety of health benefits, such as improved mood, mental health and cognition as well as increased physical activity through engaging in outdoor activities [30,31,32,33]. Busy modern lives, urban living, and changes in culture and the employment sector, mean many people are spending less time in nature [34], leading to what has been termed an ‘extinction of experience’ [35]. This disconnect is most pronounced in groups of young people [35]. Children often have knowledge of non-native, charismatic species regularly seen in the media, such as lions, tigers or pandas, but have poor understanding of local species [36,37]. Introducing nature-based citizen science into schools could help to engage young people from a diverse range of backgrounds, reversing the ‘extinction of experience’ trend and increasing connection to nature [23,38], while helping them to engage with their studies and contributing to their learning [38,39].

## 3. Camera Traps and Citizen Science

Early camera trap research was ill-suited to citizen science, as camera traps required considerable effort and technical expertise to set up, were expensive, and collected few images, which were stored on film and thus difficult to share on a large scale [40,41,42]. Two key advances in the development of camera traps made them more suitable for citizen science. First, the switch from film-based to digital storage, which became more common from 2007 onwards [1]. This enabled (i) more photographs to be collected and stored, while requiring less frequent visits to camera trap stations, and (ii) images to be viewed instantly and more easily organised and shared. The impact of this development was amplified by increased computer ownership and access to internet, which also increased the ease with which data could be stored and shared. The second characteristic was the development of a single unit camera trap that included a built-in flash and a passive infra-red detection system. Unlike active infra-red systems, which required two units to be placed opposite each other, passive IR systems require only a single unit. This feature evolved even among the film-based models, making camera traps more practical, affordable and user-friendly. Passive infra-red sensors detect the surface temperature of objects in the detection zone; when an animal enters this zone, the sensor detects the difference in surface temperatures and triggers the camera [43]. Unfortunately, this means that moving vegetation can also trigger the camera and so lead to large numbers of ‘blank’ photos, i.e., those devoid of animals. Filtering out blank images can be extremely time consuming, especially because, without close inspection, it may not always be readily apparent whether or not an image contains an animal. Despite these false triggers, modern camera traps have been found to be an effective and cost-efficient method of detecting and recording a wide range of species [1,44,45] and since 2005 [12], there has been a rapid uptake in camera trap use to answer a diversity of ecological questions [2,3]. Camera traps have also been increasingly employed as part of long-term, wide-scale monitoring programs [46]. This has led to some programs managing millions of images, including the Snapshot Serengeti project [7], the dataset collated by the Tropical Ecology Assessment and Monitoring Team (TEAM, now part of the ‘Wildlife Insights’ project, https://www.wildlifeinsights.org/) [47] and that collected by Dorji et al. in Bhutan [48]. While some projects monitor communities of species, many other surveys target a single species or taxon. Due to the large numbers of images collected, researchers may save time by focusing only on those that contain animals of interest, leaving many images as ‘bycatch’. These images might contain very useful data on many other species [49,50], but are often left unanalysed.

The development of user-friendly technology and the large volume of camera trap images being generated means there is now both the possibility and the demand for citizen science to help collect and classify data. Some of the most popular and established online citizen science projects involve observing and identifying features of interest in an image. An early but enduringly popular example is Galaxy Zoo, where participants are asked to identify features of galaxies [51]. The success of this project led to the development of Zooniverse (https://www.zooniverse.org/), an online platform currently hosting 111 active projects, with a further 70 ‘paused’, and 21 projects completed [52]. Of the active projects, 35 currently involve classification of camera trap images or videos. In addition, there are a growing number of projects hosted independently of Zooniverse, which also invite participants to contribute to camera trap research, including those on InstantWild (https://instantwild.zsl.org), MammalWeb (https://www.mammalweb.org/), eMammal (https://emammal.si.edu/), Wildlife@Home (https://csgrid.org/csg/wildlife/), Digivol (https://australianmuseum.net.au/get-involved/citizen-science/digivol/) and Wildlife Insights.

Most citizen science camera trap projects fall in the category of ‘contributory’ citizen science. This often involves presenting images collected by a researcher or organisation to the public online, and asking for species classifications. Although a small number of citizen science projects, have created their own online platforms, the large time and financial commitments and specialist knowledge needed, means that most use established platforms such as Zooniverse. This site provides a project template as well as advice on how to create a successful project. As multiple camera trap projects have already used Zooniverse there are even open source codes on GitHub (https://github.com/zooniverse/help) already available to aid with project creation and data analysis, and literature describing methods used to aggregate responses [53]. Such platforms also give access to a large, existing volunteer base, which may be particularly important if classifications are needed within a short time frame. Other existing platforms that can host camera trap images for classification by citizen scientists include CitSci.org (www.citsci.org) and iNaturalist (https://www.inaturalist.org/). iNaturalist is a popular site for sharing for photos, and gaining identifications for those images; however, it is recommended that those wishing to create successful projects on the site are themselves users, with experience of how the site works [54]. Some platforms, such as MammalWeb, are more geographically targeted and may be more effective for localised projects trying to engage local communities.

While some of the online-only citizen science camera trap projects, where participants are only involved in image classification, currently attract the largest numbers of participants, there are projects where participants can also contribute camera trap footage that they themselves have collected. There are numerous potential benefits to involving more people directly in camera trap placement including: (1) assistance with placing and monitoring large numbers of camera traps [55]; (2) ability to place traps across a wider geographic area; (3) access to private land; (4) reduced risk of theft, due to increased community engagement; and (5) financial assistance, for example through participants providing their own camera traps, or through expedition fees [25,56]. Relative to viewing and classifying images online, placing a camera trap to collect data requires a greater investment of time, energy and (if the camera trap is purchased by the participant) finances. Therefore, participants placing camera traps might be more engaged and likely to participate more regularly than those only classifying photos online. As such, they may be more likely to display benefits such as increased knowledge and changes in behaviour [57]. Actively placing a camera also involves going out into nature and could help to create a deeper affiliation with that particular place. Good camera placement involves consideration of the environment and the way it is used by wildlife, encouraging people to think about these issues. This can be particularly effective when people are collecting data in areas local to them, helping to foster a sense of place and caretaking behaviour [58,59].

## 4. Approaches Used in Camera Trapping and Citizen Science

Now that projects can be created relatively easily, and given evidence for benefits of citizen science, interest in the use of citizen science is expected to grow. However, to make sure project aims are met, important questions should be considered when designing a project; some of these are highlighted in Table 1. Many different approaches are being used to involve more of the public in setting camera traps, and the most suitable approach will depend on the research question and project aims. One example of these approaches is MammalWeb, which currently aims to record mammal distribution across the UK and parts of continental Europe. MammalWeb provides some basic guidelines but allows participants of its main project to place a camera anywhere they choose, and for any length of time, as long as this information is submitted along with any images. Participants are not automatically required to submit any classifications but may choose to classify only the images they have uploaded, or classify from a dataset including images collected by all participants. Participants may classify images even if they have not submitted any themselves and can participate as frequently, or infrequently, as they wish. The flexibility in this project is designed to encourage and enable as many people as possible to participate at whatever level they choose; however, data collection is less systematic compared to projects with predetermined camera trap sites and trapping periods.

MammalWeb also offers a platform for other organisations and individuals to pursue more specific or hypothesis driven research questions by setting up and hosting projects. There is flexibility to specify which users are able to upload images and submit classifications, and for different methodologies to be used. MammalWeb contributors have even been inspired to pursue their own avenues of mammal research through the platform, representing a high level of engagement. In one instance this research has even led to plans for designation of a new nature reserve [60]. Through the flexibility of participation offered by MammalWeb, the project could fall into any of the three types of citizen science classified above: contributory, collaborative or co-created, depending on how much a user chooses to engage. As a consequence, it may serve as a model for future citizen science projects.

EMammal is a tool to aid in management and storage of camera trap data and, although not exclusively for projects involving citizen science, it provides a platform for scientists to invite the public to participate in their research. The site hosts over 110 projects in 22 countries. Some of these invite citizen scientists to help collect data by placing camera traps in the field and classifying camera trap footage. eMammal advises citizen scientists to place cameras randomly, but exact study design will be described by the individual project leader [61]. This creates targeted data collection for answering specific research questions, enabling participation by those available and able to follow that project design at the time (i.e., participants must be able to visit a specific study area during a particular time period). eMammal also supports camera trapping in schools, providing resources for teachers and encouraging pupils to design their own research questions and collect data using camera traps, which is then validated by experts on eMammal [39].

Another project that offers a platform for citizen science projects is the ‘WildBook’ (www.wildbook.org) initiative. Like eMammal, WildBook, on its own, is not just a citizen science project, but provides a framework that can be used to support a variety of projects. WildBook is a piece of software designed to support projects using images collected by citizen scientists and integrating AI into the image classification process. As a tool, WildBook supports projects where the mass participation element comes through submission of photos, and classification of these images is then done through AI and/or by project leaders. Many projects focus not just on identification of particular species, but on identification of individuals. Consequently, they may ask for only images of a particular species to be submitted. Images can be taken manually [62] but some projects can combine images taken manually with those taken with a camera trap [63]. While this project differs from the others in that it does not focus exclusively on camera trap images, it is a good example of the shift in format, where human participation is through submission of photographs, and the burden of classification is borne substantially by AI.

The Wildlife Insights project also offers a platform where individuals or organisations can upload image data and gain AI assisted classifications for that data [64]. In this case, the platform is specifically designed for working with camera trap image data. This data is then aggregated to form a global database. The platform also contains data sharing and data analysis modules, in order to enable sharing of wildlife data and assist with better management of wildlife populations [64]. While Wildlife Insights is not specifically a citizen science project, its open nature means that members of the public that are collecting camera trap images for their own interest or research could add their data to the platform, thus contributing to global wildlife records. Data can also be downloaded and used for an individual’s own research.

Another format for engaging the public in camera trapping research is through harnessing the popularity of ecotourism. For example, the EarthWatch Institute (https://earthwatch.org/) runs over 60 different expeditions in countries across the world, attracting over 2000 participants a year [56]. Participants pay to join the expedition for a short period of time, providing financial support for the research. Where these expeditions involve camera trapping, participants can assist in both camera placement and classification of images collected, while being supervised and supported by researchers. Data collection is often hypothesis driven and structured towards specific research goals, and has already been used to contribute towards research on a variety of topics including large carnivore density and population dynamics, and impacts of cattle on forest fauna [65,66,67].

## 5. Practical Considerations for the Use of Camera Trapping and Citizen Science

Researchers may be deterred from engaging in citizen science by concerns regarding data quality. Due to this concern, data collected by citizen scientists are not always as highly valued within the scientific community [68,69,70,71]. In some cases, there is justification for these concerns; however, for most projects, data quality can be assured by adopting a good study design and training volunteers, and by the use of vouchers [72,73]. A voucher is a physical or tangible reference that can be used to verify a data point [72]. Photographs are a common example of this, as they can serve as evidence of a particular species or event that can be checked if the datum is questioned. Camera trapping is particularly well suited to this as the images or videos produced can be used as vouchers. The camera trap images can be used to verify classification accuracy, as well as whether the camera trap itself was correctly positioned and, thus, if the data collected from that unit are valid.

Using a few highly trained experts to verify camera trap image classifications is one approach to maximise accuracy, but is time consuming and would not make best use of citizen scientists’ efforts. Alternatively, multiple project participants can view and classify each image, and the results of individual classifications can be aggregated to create a final single classification. This provides a probability and degree of confidence that the final classification being reached is the correct one, and can be used to reduce the likelihood of errors in the data set. Consensus algorithms have already been used to reach a final classification for data sets managed by Snapshot Serengeti [53] and MammalWeb [6], with both projects concluding that a high level of confidence in classifications can be reached by aggregating responses. Assessments of classification accuracy concluded that, on average, 10 classifications were needed per image to reach a 95% accuracy level for Snapshot Serengeti [53], and a >99% accuracy on MammalWeb [6], although both studies found this varied among species. However, with over three million image sequences on active Zooniverse projects alone [52], the demand for sufficient classifications to reach a confident consensus may outstrip capacity, such that projects may find themselves competing to attract enough participants.

## 6. Integrating AI into Camera Trap and Citizen Science Work Flows

The use of computer vision, the subset of AI associated with visual data, to automate the classification of camera trap images is a technique rapidly gaining interest [79,80] and can be used as an alternative, or in conjunction with, citizen science. Classification through AI can use neural networks, which are mathematical algorithms that map an input object to an output. An example of a relevant input object is an image, with the output being a classification for that image. The optimisation of one of these algorithms for a task is often referred to as training and requires labelled data corresponding to the input objects used by the final network to make a prediction. In the context of camera trapping, this would mean having a large reference collection of pre-classified, or ‘labelled’, images. During training, the algorithms are iteratively updated to minimise the loss function. The loss function calculates how different the predicted output of the network (the animal the network calculates as most probable) is to the training label (the actual animal in the image). This process continues iteratively for either a predefined length of time or until the desired performance (e.g., a threshold species classification accuracy) is reached on the validation dataset. Implementing this training procedure, known as Deep Learning, can be achieved in a number of ways depending upon technical knowledge such as coding ability. With coding knowledge, free open source solutions are available; alternatively, packaged software is available but costs money. The time scale and complexity of the problem will undoubtedly impact the financial investment required. Deep Learning is a rapidly developing commercial and academic field, with usability coming to the forefront. Improved ease of use, increases in computational power, and the decreasing price of the graphics processing units (GPUs) used for training, are making the use of AI a realistic and practical option for an ever-widening audience.

With the advent of Deep Learning and the improved image analysis performance associated with convolutional neural networks (CNNs), robust and accurate analysis of ecological images is now possible [81,82,83]. However, large diverse training sets are required, and the use of computer vision may have been limited thus far by the significant amount of time and money needed to create these trainings sets. A solution to this problem is emerging with the help of platforms such as Zooniverse and Wildlife Insights, which are creating large datasets suitable for training. Both of these platforms incorporate citizen science to varying extents. An instance of the effective integration of citizen scientist in deep learning is Wildlife@Home, where citizen science classifications were used to train neural networks that help to analyse bird populations [84]. The growing set of citizen science classified datasets, in addition to several research teams’ datasets [85,86], are also being used to create software packages to aid ecological projects. Examples of this software are the ‘R’ package ‘Machine Learning for Wildlife Image Classification’ [4] and the pretrained networks in ‘ClassifyMe’ [87]. Where there is enough training data, the performance of CNNs targeted at camera trap image classification is promising (Table 2).

Classification accuracy is not 100% in any study, although a number of studies were able to achieve in excess of 90% accuracy when a large amount of training data were available (Table 2). Nevertheless, for studies with small training data sets relative to the problem, accuracy may drop significantly [81]. Even where accuracy is high there is still an issue of generalisability, not captured in this broad performance metric, where a model performs worse on data on which it has not been specifically trained [85]. For example, performance might be substantially lower when a network trained to recognise a particular species is given the task of doing so against a novel habitat background. Such a problem is likely to be common with camera-trapping, since new camera locations might be added to an existing array or additional projects established at new geographic locations. In such instances, projects may need to create new or enhance existing training sets, undermining the potential benefits of the AI approach since significant effort is still needed in manual classifications. Furthermore, the large-scale studies for which very high accuracies are achieved are not representative of all conservation or research efforts, many of which generate smaller data sets. Creating a training set of labelled camera trap images is laborious and, for small-scale conservation efforts, could be an impractical and inefficient process. Indeed, training set requirements could necessitate a significant proportion of camera trap images to be labelled to achieve satisfactory classification accuracy. If only small training sets are available, it is not yet likely that a CNN can be deployed in a fully autonomous system (without human intervention) for classifying animals within images.

A semi-autonomous approach (with limited human intervention) is the natural solution to this and is well suited to being incorporated into researcher and citizen science workflows. This reduces the strain on researchers and small projects that cannot gather enough classifications for consensus accuracy in a reasonable timeframe [5]. This semi-autonomy can occur by setting a confidence threshold on the output of network classifications to accept only those with the desired confidence. Classifications over the desired confidence threshold are assumed to be correct and do not need to be verified by a human. Norouzzadeh et al. found that citizen science accuracy was 96.6% for the Snapshot Serengeti dataset of animals in the in Serengeti National Park, Tanzania. Thresholding neural network classifications to the same value of 96.6% would allow for 99.3% of the data in this project to be automatically processed [5]. Classifications not satisfying the desired threshold are more likely to contain errors and could be flagged for classification by a human. If the number of images requiring manual classification is small, this task could be completed by researchers. In many cases, however, this stage could benefit from the involvement of citizen scientists.

As described earlier, multiple citizen scientist classifications can be combined and a consensus classification algorithm used to reach a final classification [6,53]. A way of integrating AI and citizen science classifications would be to use the AI classification as another vote which could be weighted based on confidence in the classification in this process. This would involve showing all camera trap footage to both the citizen scientists and the neural network, but would reduce the number of human classifications needed per image in order to reach a consensus classification.

One approach to achieving a consensus classification workflow is to perform a ‘cascade filtering’ workflow with a series of nested consensus classification stages [90]. This method of cascade filtering was trialled by Willi et al. using AI and citizen science classifications on camera trap data sets from the Zooniverse project ‘Camera CATalogue’, which aims to capture data on large cat species [90]. The ‘cascade filtering’ workflow was comprised of multiple easier binary classifications, e.g., does the image contain an object or not, followed by whether the image contains a vehicle or not, and finally a species classification. This approach reduced the number of people required to agree with the model before the image was retired at each level, allowing data sets to be classified more quickly. This was demonstrated in a trial on a Zooniverse project where human effort was reduced by 43% [90]. Approaches that integrate AI with citizen science thus offer significant potential to efficiently classify camera trapping data sets.

It is not necessary to use cascade filtering to achieve consensus classifications. This could be done on a single tier, with citizen scientists and AI both providing a species ID. However, there has been little published work on applying this method of achieving a consensus classification. We see great potential in this method and believe it merits further attention. Trialling different methods, such as those described above, on diverse data sets will help highlight which are most effective for future use.

## 7. Future Directions and Conclusions

Currently camera trap projects may be constrained by the time needed to classify footage. This can lead to some images remaining unidentified and unused, and may significantly undermine the significance and potential of data sets. Citizen science and AI have emerged as two potential solutions to this constraint, although independently both have limitations that suggest that neither represent a complete solution in isolation. We believe that the integration of AI and citizen science offers significant long-term promise and initial results from some recent projects suggests it is already starting to achieve this potential. We envisage four main formats in which a camera trap project might combine citizen science with AI:

1. Classifications of camera trap footage are submitted by citizen scientists, thus creating a labelled data set which can be used to train a neural network, which can then be used to classify future footage from that project.
2. Use of a combination of AI and citizen science classifications to reach a consensus. All images could be shown to both the neural network and citizen scientists, with the network representing an extra ‘vote’ and therefore reducing the number of human classifications needed to reach a consensus. Alternatively, greater efficiency could be attained by first obtaining AI classifications for a data set, then presenting only outputs of AI classifications with low accuracy confidence to citizen scientists for confirmation.
3. Pre-screening of data using AI to filter out blank footage, or species of interest, with citizen scientists then examining the resultant footage to extract further information, such as animal behaviour or identifying recognisable individuals.
4. Camera traps are placed in the field and monitored by citizen scientists. Resultant footage is then classified using AI.

These formats are not mutually exclusive and a project may progress from one format to another over time. For example, using citizen scientist classifications to train a neural network in the beginning, but then later focussing participant efforts elsewhere such as on the placement of more cameras, or gathering additional details from classified footage. As the technology behind AI develops further, it is likely that it will be used to classify a growing proportion of camera trap images being collected. Yet even with the development of AI, the need for citizen science will not be lost. AI cannot provide the engagement benefits associated with citizen science, the value of which should not be dismissed. This is particularly important for studies that require people to not just classify images, but to assist in other elements of the research process including camera placement and servicing. Furthermore, AI—while able to assist in classification of species in an image—cannot perform the full range of activities of a human participant. Camera trap footage can provide more information than simply the presence of an animal and is increasingly being used in behavioural studies [91,92,93,94]. While some research uses AI to recognise animal behaviours, this has been less successful than the simple identification of a species [5]. Here, we see potential to combine AI with human efforts, as AI could be used to identify and filter out a species of interest for a particular study, thus greatly reducing the human workload. Such footage could be shown to human observers able to extract further detail, such as behaviours or the number of animals in an image.

Overall, integration of citizen science and AI technology into camera trapping research can be used to help maximise the amount of data that can be collected and processed efficiently, while simultaneously engaging and informing people about the natural world and its value. Although AI, citizen science, or both, may not be suitable for all projects, we believe they should be given serious consideration and integrated wherever possible, as numerous potential benefits can be realised.

## Figures and Tables

**Table 1:** Considerations for citizen science camera trap project design.

Planning Stage	Considerations	Suggestions
Project Aims	What are the desired outcomes of the project with regard to research, engagement, education and other social benefits?	Many citizen science projects will have multiple aims with regard to data collection and other social and engagement benefits. While it is possible to achieve multiple aims, some compromise may be needed. Decide on the priorities for your project at the start, as this will help develop the best methodologies to achieve your aims.
Research Question	Do you have an existing question or are you aiming to work with a community in order to develop questions collaboratively?	If you have an existing question, consider how it might be relevant or interesting to the community you are trying to engage. If you plan to work with a community to develop a question, make sure you allow time to develop a good relationship with the community and try to include as many different people and perspectives in the planning process as possible.
Methodology	Does your methodology enable members of the public to contribute meaningfully?	If contributors need to learn and follow a methodology in a short period of time, it needs to be as clear and simple as possible. If specialist equipment is required, consider whether you can provide this equipment. Provide guidelines to those placing camera traps, such as recommended settings and camera positions. Have data quality checks in place, such as assessments of camera placement and footage submitted, so that feedback can be provided to participants to help them provide meaningful data.
	Do camera traps need to be set out in specific locations or formats in the field, and will these be accessible to the public?	Engaging members of the public may help to open up access to private land owned by participants, but it is also important to ensure participants understand the privacy and ethical issues around camera trapping and ask permission before placing cameras on private land owned by others. Consider how safe it is for participants to visit remote sites and provide a risk assessment and health and safety guidelines. Consider organising group trips, or providing a platform for participants to communicate and work together. If camera traps need to be set in precise locations, plan how to communicate these locations safely without advertising them to people outside the project, minimising risk of theft and vandalism.
	Can citizen science be used to assist in image classification, and how can accuracy be ensured?	Image classification is a popular way of engaging people in citizen science camera trapping and it is important to be able to trust the classifications provided. To ensure high levels of accuracy, expert verification can be used, or multiple classifications per image acquired from the general public, which can then be aggregated to reach a consensus classification [6,7].
	Will additional training of citizen scientists be needed and how can you provide this?	Training requirements will vary depending on the stages of the research process in which people will participate. Instruction sheets or instructional videos can be provided online. Online resources can reach larger audiences, so they are good for large scale projects. Alternatively, or in addition to this, workshops and training days could be used to give more in-depth practical training. Another model for ensuring correct data collection is for it to be undertaken with expert supervision [67], although this does mean data collection is constrained by expert availability. Training roles could be outsourced to experienced participants and additional training provided to small groups who can then train others. Education, or the desire to learn something new, can motivate participation; hence, providing learning opportunities can also help to engage participants [74].
Engagement	Can you recruit enough people to participate and how will you engage people so that they are motivated to work on your project?	Regular communication and project feedback [75], use of social media [76], gamification [77] and providing opportunities for social interactions within a participant community [27,78] have all been shown to help increase awareness, motivation or participation. Where large numbers of classifications are needed, integrating use of artificial intelligence may help to alleviate workload (see main text).
	Who are you trying to engage? What barriers to participation might there be, e.g., not owning a camera trap or computer, or not having access to internet?	Get to know your focal community so that potential barriers can be taken into consideration when designing a methodology. Some equipment could be lent to individuals or communities.

**Table 2:** Examples of convolutional neural networks (CNNs) use to predict species present in camera trap images. Here, accuracy is defined as the percentage of correct predictions by the network. Accuracy figures refer to the top-performing model, or ensemble of models, from each study. Balanced refers to the fact that there is the same number of images in each species class. No object segmentation or detection was used.

Number of Different Species	Number of Images in the Dataset	Taxa Location	Species Classification Accuracy (%)	Reference
30	3,367,383	USA	98	Tabak et al., 2019 [4]
48	3,200,000	Serengeti	93.8	Norouzzadeh et al., 2018 [5]
26	26,000 (Balanced)	Serengeti	67 ^1^	Gomez Villa et al., 2017 [88]
6	62,853	Australia	84.4	Nguyen et al., 2017 [89]
31	300,000	Various	91.4	Willi et al., 2019 [90]
20	23,876	North America	38.3	Chen et al., 2014 [81]

^1^ Exact number not reported; estimated from a graph.

## References

[1] Wearn O.R., Glover-Kapfer P. (2019). Snap happy: Camera traps are an effective sampling tool when compared with alternative methods. R. Soc. Open Sci..

[2] Meek P.D., Ballard G.A., Vernes K., Fleming P.J.S. (2015). The history of wildlife camera trapping as a survey tool in Australia. Aust. Mammal..

[3] Glover-Kapfer P., Soto-Navarro C.A., Wearn O.R. (2019). Camera-trapping version 3.0: Current constraints and future priorities for development. Remote Sens. Ecol. Conserv..

[4] Tabak M.A., Norouzzadeh M.S., Wolfson D.W., Sweeney S.J., Vercauteren K.C., Snow N.P., Halseth J.M., Di Salvo P.A., Lewis J.S., White M.D. (2019). Machine learning to classify animal species in camera trap images: Applications in ecology. Methods Ecol. Evol..

[5] Norouzzadeh M.S., Nguyen A., Kosmala M., Swanson A., Palmer M., Packer C., Clune J. (2018). Automatically identifying, counting, and describing wild animals in camera-trap images with deep learning. Proc. Natl. Acad. Sci. USA.

[6] Hsing P.-Y., Bradley S., Kent V.T., Hill R.A., Smith G.C., Whittingham M.J., Cokill J., Crawley D., Stephens P.A., Stephens P.A. (2018). Economical crowdsourcing for camera trap image classification. Remote Sens. Ecol. Conserv..

[7] Swanson A., Kosmala M., Lintott C., Simpson R., Smith A., Packer C. (2015). Snapshot Serengeti, high-frequency annotated camera trap images of 40 mammalian species in an African savanna. Sci. Data.

[8] Rovero F., Zimmermann F. (2016). Camera Trapping for Wildlife Research.

[9] Brower M. (2008). George Shiras and the circulation of wildlife photography. Hist. Photogr..

[10] Griffiths M., van Schaik C.P. (1993). Camera trapping: A new tool for the study of elusive rain forest animals. Trop. Biodivers..

[11] Rovero F., Tobler M., Sanderson J., Samyn Y., Vandenspiegel D., Degreef J. (2010). Camera trapping for inventorying terrestrial vertebrates. Manual on Field Recording Techniques and Protocols for All Taxa Biodiversity Inventories.

[12] Rovero F., Zimmermann F., Berzi D., Meek P. (2013). “Which camera trap type and how many do I need?” A review of camera features and study designs for a range of wildlife research applications. Hystrix.

[13] Meek P.D., Pittet A. (2012). User-based design specifications for the ultimate camera trap for wildlife research. Wildl. Res..

[14] OED (2019). Citizen, n. and adj.. https://www.oed.com/view/Entry/33513.

[15] Masters K., Oh E.Y., Cox J., Simmons B., Lintott C., Graham G., Greenhill A., Holmes K. (2016). Science Learning via Participation in Online Citizen Science. J. Sci. Commun..

[16] Geoghegan H., Dyke A., Pateman R., West S., Everett G. (2016). Understanding Motivations for Citizen Science. Final Report on Behalf of the UK Environmental Observation Framework (UKEOF).

[17] Haklay M., Sui D.Z., Elwood S., Goodchild M.F. (2013). Citizen Science and Volunteered Geographic Information-overview and typology of participation. Crowdsourcing Geogrpahic Knowledge: Volunteered Geogrpahic Information (VGI) inTheory and Practice.

[18] Eitzel M.V., Cappadonna J.L., Santos-Lang C., Duerr R.E., Virapongse A., West S.E., Kyba C.C.M., Bowser A., Cooper C.B., Sforzi A. (2017). Citizen Science Terminology Matters: Exploring Key Terms. Citiz. Sci. Theory Pract..

[19] Shirk J.L., Ballard H.L., Wilderman C.C., Phillips T., Wiggins A., Jordan R., McCallie E., Minarchek M., Lewenstein B.V., Krasny M.E. (2012). Public Participation in Scientific Research: A Framework for Deliberate Design. Ecol. Soc..

[20] Bonney R., Ballard H.L., Jordan R., McCallie E., Phillips T., Shirk J., Wilderman C.C. (2009). Public Participation in Scientific Research: Defining the Field and Assessing its Potential for Informal Science Education Caise Center for Advancement of Informal Science Education. A CAISE Inquiry Group Report.

[21] Roy H.E., Pocock M.J.O., Preston C.D., Roy D.B., Savage J., Tweddle J.C., Robinson L.D. (2012). Understanding Citizen Science and Environmental Monitoring.

[22] Forrester T.D., Baker M., Costello R., Kays R., Parsons A.W., McShea W.J. (2017). Creating advocates for mammal conservation through citizen science. Biol. Conserv..

[23] Schuttler S.G., Sorensen A.E., Jordan R.C., Cooper C., Shwartz A. (2018). Bridging the nature gap: Can citizen science reverse the extinction of experience?. Front. Ecol. Environ..

[24] Dayer A.A., Rosenblatt C., Bonter D.N., Faulkner H., Hall R.J., Hochachka W.M., Phillips T.B., Hawley D.M. (2019). Observations at backyard bird feeders influence the emotions and actions of people that feed birds. People Nat..

[25] Toomey A.H., Domroese M.C. (2013). Can citizen science lead to positive conservation attitudes and behaviors. Hum. Ecol. Rev..

[26] Lewandowski E.J., Oberhauser K.S. (2017). Butterfly citizen scientists in the United States increase their engagement in conservation. Biol. Conserv..

[27] Curtis V. (2015). Motivation to Participate in an Online Citizen Science Game: A Study of Foldit. Sci. Commun..

[28] Domroese M.C., Johnson E.A. (2017). Why watch bees? Motivations of citizen science volunteers in the Great Pollinator Project. Biol. Conserv..

[29] Hobbs S.J., White P.C.L. (2012). Motivations and barriers in relation to community participation in biodiversity recording. J. Nat. Conserv..

[30] O’brien L., Townsend M., Ebden M. (2010). Doing Something Positive’: Volunteers’ Experiences of the Well-Being Benefits Derived from Practical Conservation Activities in Nature Article in International Journal of Voluntary and Nonprofit Organizations. Vountas.

[31] Cox D.T.C., Shanahan D.F., Hudson H.L., Fuller R.A., Anderson K., Hancock S., Gaston K.J. (2017). Doses of nearby nature simultaneously associated with multiple health benefits. Int. J. Environ. Res. Public Health.

[32] Engemann K., Pedersen C.B., Arge L., Tsirogiannis C., Mortensen P.B., Svenning J.-C. (2019). Residential green space in childhood is associated with lower risk of psychiatric disorders from adolescence into adulthood. Proc. Natl. Acad. Sci. USA.

[33] Bratman G.N., Daily G.C., Levy B.J., Gross J.J. (2015). The benefits of nature experience: Improved affect and cognition. Landsc. Urban Plan..

[34] Cox D.T.C., Hudson H.L., Shanahan D.F., Fuller R.A., Gaston K.J. (2017). The rarity of direct experiences of nature in an urban population. Landsc. Urban Plan..

[35] Soga M., Gaston K.J. (2016). Extinction of experience: The loss of human-nature interactions. Front. Ecol. Environ..

[36] Genovart M., Tavecchia G., Enseñat J.J., Laiolo P. (2013). Holding up a mirror to the society: Children recognize exotic species much more than local ones. Biol. Conserv..

[37] Tanner D. (2010). Fifth graders’ knowledge, attitudes, and behavior toward habitat loss and landscape fragmentation. Hum. Dimens. Wildl..

[38] Ballard H.L., Dixon C.G.H., Harris E.M. (2017). Youth-focused citizen science: Examining the role of environmental science learning and agency for conservation. Biol. Conserv..

[39] Schuttler S.G., Sears R.S., Orendain I., Khot R., Rubenstein D., Rubenstein N., Dunn R.R., Baird E., Kandros K., O’Brien T. (2018). Citizen Science in Schools: Students Collect Valuable Mammal Data for Science, Conservation and Community Engagement. Bioscience.

[40] Karanth K.U. (1995). Estimating Tiger Panthera tigris Populations from Camera-Trap Data Using Capture-Recapture Models. Biol. Conserv..

[41] van Schaik C.P., Griffiths M. (1996). Activity Periods of Indonesian Rain Forest Mammals. Biotropica.

[42] Seydack A.H.W. (1984). Application of a photo-recording device in the census of larger rain-forest mammals. S. Afr. J. Wildl. Res..

[43] Welbourne D.J., Claridge A.W., Paull D.J., Lambert A. (2016). How do passive infrared triggered camera traps operate and why does it matter? Breaking down common misconceptions. Remote Sens. Ecol. Conserv..

[44] Welbourne D.J., MacGregor C., Paull D., Lindenmayer D.B. (2015). The effectiveness and cost of camera traps for surveying small reptiles and critical weight range mammals: A comparison with labour-intensive complementary methods. Wildl. Res..

[45] Kämmerle J.-L., Corlatti L., Harms L., Storch I. (2018). Methods for assessing small-scale variation in the abundance of a generalist mesopredator. PLoS ONE.

[46] Steenweg R., Hebblewhite M., Kays R., Ahumada J., Fisher J.T., Burton C., Townsend S.E., Carbone C., Rowcliffe J.M., Whittington J. (2017). Scaling-up camera traps: Monitoring the planet’s biodiversity with networks of remote sensors. Front. Ecol. Environ..

[47] Rovero F., Ahumada J. (2017). The Tropical Ecology, Assessment and Monitoring (TEAM) Network An early warning system for tropical rain forests. Sci. Total Environ..

[48] Dorji S., Rajaratnam R., Vernes K. (2019). Mammal richness and diversity in a Himalayan hotspot: The role of protected areas in conserving Bhutan’s mammals. Biodivers. Conserv..

[49] Scotson L., Fredriksson G., Ngoprasert D., Wong W.-M., Fieberg J. (2017). Projecting range-wide sun bear population trends using tree cover and camera-trap bycatch data. PLoS ONE.

[50] Thapa K., Kelly M.J., Pradhan N.M.B. (2019). Elephant (*Elephas maximus*) temporal activity, distribution, and habitat use patterns on the tiger’s forgotten trails across the seasonally dry, subtropical, hilly Churia forests of Nepal. PLoS ONE.

[51] Lintott C.J., Schawinski K., Slosar A., Land K., Bamford S., Thomas D., Raddick M.J., Nichol R.C., Szalay A., Andreescu D. (2008). Galaxy Zoo: Morphologies derived from visual inspection of galaxies from the Sloan Digital Sky Survey. Mon. Not. R. Astron. Soc..

[52] Zooniverse. https://www.zooniverse.org/.

[53] Swanson A., Kosmala M., Lintott C., Packer C. (2016). A generalized approach for producing, quantifying, and validating citizen science data from wildlife images. Conserv. Biol..

[54] INaturalist Managing Projects. https://www.inaturalist.org/pages/managing-projects.

[55] Barrueto M., Ford A.T., Clevenger A.P. (2014). Anthropogenic effects on activity patterns of wildlife at crossing structures. Ecosphere.

[56] Chandler M., See L., Copas K., Bonde A.M.Z., López B.C., Danielsen F., Legind J.K., Masinde S., Miller-Rushing A.J., Newman G. (2017). Contribution of citizen science towards international biodiversity monitoring. Biol. Conserv..

[57] Jones M., Riddell K., Morrow A. (2013). The Impact of Citizen Science Activities on Participant Behaviour and Attitude.

[58] Evans C. (2005). The Neighborhood Nestwatch Program: Participant Outcomes of a Citizen-Science Ecological Research Project. Conserv. Educ..

[59] Haywood B.K., Parrish J.K., Dolliver J. (2016). Place-based and data-rich citizen science as a precursor for conservation action. Conserv. Biol..

[60] McKie R. How an Army of “Citizen Scientists” Is Helping Save Our Most Elusive Animals. Guardian. https://www.theguardian.com/environment/2019/jul/28/britain-elusive-animals-fall-into-camera-trap-citizen-scientist.

[61] Parsons A.W., Forrester T., Baker-Whatton M.C., Mcshea W.J., Rota C.T., Schuttler S.G., Millspaugh J.J., Kays R. (2018). Mammal communities are larger and more diverse in moderately developed areas. Elife.

[62] Paxton A.B., Blair E., Blawas C., Fatzinger M.H., Marens M., Holmberg J., Kingen C., Houppermans T., Keusenkothen M., McCord J. (2019). Citizen science reveals female sand tiger sharks (Carcharias taurus) exhibit signs of site fidelity on shipwrecks. Ecology.

[63] Koivuniemi M., Kurkilahti M., Niemi M., Auttila M., Kunnasranta M. (2019). A mark–recapture approach for estimating population size of the endangered ringed seal (*Phoca hispida* saimensis). PLoS ONE.

[64] Ahumada J.A., Fegraus E., Birch T., Flores N., Kays R., O’Brien T.G., Palmer J., Schuttler S., Zhao J.Y., Jetz W. (2019). Wildlife Insights: A Platform to Maximize the Potential of Camera Trap and Other Passive Sensor Wildlife Data for the Planet. Environ. Conserv..

[65] Sollmann R., Furtado M.M., Gardner B., Hofer H., Jácomo T.A., Tôrres N.M., Silveira L. (2011). Improving density estimates for elusive carnivores: Accounting for sex-specific detection and movements using spatial capture-recapture models for jaguars in central Brazil. Biol. Conserv..

[66] Williams S.T., Williams K.S., Lewis B.P., Hill R.A. (2017). Population dynamics and threats to an apex predator outside protected areas: Implications for carnivore management. R. Soc. Open Sci..

[67] Eaton D.P., Keuroghlian A., do Santos M.C.A. (2017). Citizen scientists help unravel the nature of cattle impacts on native mammals and birds visiting fruiting trees in Brazil’s southern Pantanal. Biol. Conserv..

[68] Catlin-Groves C.L. (2012). The Citizen Science Landscape: From Volunteers to Citizen Sensors and Beyond. Int. J. Zool..

[69] Dickinson J.L., Zuckerberg B., Bonter D.N. (2010). Citizen Science as an Ecological Research Tool: Challenges and Benefits. Annu. Rev. Ecol. Evol. Syst..

[70] Ellwood E.R., Crimmins T.M., Miller-Rushing A.J. (2017). Citizen science and conservation: Recommendations for a rapidly moving field. Biol. Conserv..

[71] Burgess H.K., DeBey L.B., Froehlich H.E., Schmidt N., Theobald E.J., Ettinger A.K., HilleRisLambers J., Tewksbury J., Parrish J.K. (2017). The science of citizen science: Exploring barriers to use as a primary research tool. Biol. Conserv..

[72] Kosmala M., Wiggins A., Swanson A., Simmons B. (2016). Assessing data quality in citizen science. Front. Ecol. Environ..

[73] MacKenzie C.M., Murray G., Primack R., Weihrauch D. (2017). Lessons from citizen science: Assessing volunteer-collected plant phenology data with Mountain Watch. Biol. Conserv..

[74] Ganzevoort W., van den Born R.J.G., Halffman W., Turnhout S. (2017). Sharing biodiversity data: Citizen scientists’ concerns and motivations. Biodivers. Conserv..

[75] Rotman D., Hammock J., Preece J., Hansen D., Boston C., Bowser A., He Y. (2014). Motivations Affecting Initial and Long-Term Participation in Citizen Science Projects in Three Countries. Proceedings.

[76] Robson C., Hearst M.A., Kau C., Pierce J. Comparing the Use of Social Networking and Traditional Media Channels for Promoting Citizen Science Human Factors. Proceedings of the 2013 Conference on Computer Supported Cooperative Work.

[77] Bowser A., Hansen D., He Y., Boston C., Reid M., Gunnell L., Preece J. Using gamification to inspire new citizen science volunteers. Proceedings of the First International Conference on Gameful Design, Research, and Applications—Gamification’13.

[78] Tinati R., Luczak-Roesch M., Simperl E., Hall W. (2017). An investigation of player motivations in Eyewire, a gamified citizen science project. Comput. Human Behav..

[79] Weinstein B.G. (2018). A computer vision for animal ecology. J. Anim. Ecol..

[80] Wäldchen J., Mäder P. (2018). Machine learning for image based species identification. Methods Ecol. Evol..

[81] Chen G., Han T.X., He Z., Kays R., Forrester T. Deep convolutional neural network based species recognition for wild animal monitoring. Proceedings of the 2014 IEEE International Conference on Image Processing, ICIP 2014.

[82] Qin H., Li X., Liang J., Peng Y., Zhang C. (2016). DeepFish: Accurate underwater live fish recognition with a deep architecture. Neurocomputing.

[83] Trnovszky T., Kamencay P., Orjesek R., Benco M., Sykora P. (2017). Animal recognition system based on convolutional neural network. Adv. Electr. Electron. Eng..

[84] Bowley C., Mattingly M., Barnas A., Ellis-Felege S., Desell T. (2019). An Analysis of Altitude, Citizen Science and a Convolutional Neural Network Feedback Loop on Object Detection in Unmanned Aerial Systems. J. Comput. Sci..

[85] Beery S., Van Horn G., Perona P. Recognition in Terra Incognita. Proceedings of the European Conference on Computer Vision (ECCV).

[86] Van Horn G., Aodha O., Song Y., Cui Y., Sun C., Shepard A., Adam H., Perona P., Belongie S. The iNaturalist Species Classification and Detection Dataset. Proceedings of the IEEE Computer Society Conference on Computer Vision and Pattern Recognition (CVPR).

[87] Falzon G., Lawson C., Cheung K.-W., Vernes K., Ballard G.A., Fleming P.J.S., Glen A.S., Milne H., Mather-Zardain A.T., Meek P.D. (2020). ClassifyMe: A field-scouting software for the identification of wildlife in camera trap. Animals.

[88] Gomez Villa A., Salazar A., Vargas F. (2017). Towards automatic wild animal monitoring: Identification of animal species in camera-trap images using very deep convolutional neural networks. Ecol. Inform..

[89] Nguyen H., Maclagan S.J., Nguyen T.D., Nguyen T., Flemons P., Andrews K., Ritchie E.G., Phung D. Animal recognition and identification with deep convolutional neural networks for automated wildlife monitoring. Proceedings of the 2017 International Conference on Data Science and Advanced Analytics, DSAA 2017.

[90] Willi M., Pitman R.T., Cardoso A.W., Locke C., Swanson A., Boyer A., Veldthuis M., Fortson L. (2019). Identifying animal species in camera trap images using deep learning and citizen science. Methods Ecol. Evol..

[91] Caravaggi A., Banks P.B., Burton A.C., Finlay C.M.V., Haswell P.M., Hayward M.W., Rowcliffe M.J., Wood M.D. (2017). A review of camera trapping for conservation behaviour research. Remote Sens. Ecol. Conserv..

[92] Orta-Martínez M., Rosell-Melé A., Cartró-Sabaté M., O’Callaghan-Gordo C., Moraleda-Cibrián N., Mayor P. (2018). First evidences of Amazonian wildlife feeding on petroleum-contaminated soils: A new exposure route to petrogenic compounds?. Environ. Res..

[93] Kalan A.K., Hohmann G., Arandjelovic M., Boesch C., McCarthy M.S., Agbor A., Angedakin S., Bailey E., Balongelwa C.W., Bessone M. (2019). Novelty Response of Wild African Apes to Camera Traps. Curr. Biol..

[94] Vernes K., Jarman P. (2014). Long-nosed potoroo (Potorous tridactylus) behaviour and handling times when foraging for buried truffles. Aust. Mammal..

